# CARD15 Gene Polymorphisms Are Associated with Tuberculosis Susceptibility in Chinese Holstein Cows

**DOI:** 10.1371/journal.pone.0135085

**Published:** 2015-08-05

**Authors:** Youtao Wang, Shengkui Wang, Tong Liu, Wenji Tu, Wengui Li, Guodong Dong, Cong Xu, Bo Qin, Kaihua Liu, Jie Yang, Jun Chai, Xianwei Shi, Yifang Zhang

**Affiliations:** 1 College of Animal Science and Technology, Yunnan Agricultural University, Kunming, Yunnan Province, China; 2 College of Hydraulic and Architectural Engineering, Yunnan Agricultural University, Kunming, Yunnan Province, China; 3 Center for Animal Disease Control and Prevention of Yunnan Province, Kunming, Yunnan Province, China; 4 Center for Animal Disease Control and Prevention of Yuxi City, Yuxi, Yunnan Province, China; China Agricultural Univeristy, CHINA

## Abstract

Bovine tuberculosis (BTB) is a significant veterinary and financial problem in many parts of the world. Associations between specific host genes and susceptibility to mycobacterial infections, such as tuberculosis, have been reported in several species. The objective of this study was to identify and evaluate the relationship of single-nucleotide polymorphisms (SNPs) in the CARD15 gene with susceptibility to BTB in Chinese Holstein cows. DNA samples from 201 Chinese Holstein cows (103 cases and 98 controls) were collected from Kunming City, Yuxi City, and Dali City in China. SNPs in the CARD15 gene were assessed using polymerase chain reaction (PCR) and restriction fragment length polymorphism-polymerase chain reaction (RFLP-PCR). Case-control association testing and statistical analysis identified six SNPs associated with susceptibility to BTB in Chinese Holstein cows. The frequency of genotypes C/T, A/G, A/G, A/G, C/T, and A/G in E4 (-37), 208, 1644, 1648, 1799, and E10 (+107), respectively, was significantly higher in cases than in controls, and also the alleles C, A, A, G, T, and A, respectively, were associated with a greater relative risk in cases than in controls. The distribution of two haplotypes, TGGACA and CAGACA, was significantly different between cases and controls. Overall, this case-control study suggested that E4 (-37)(C/T), 208(A/G), 1644(A/G), 1648(A/G), 1799(C/T), and E10 (+107)(A/G) in the CARD15 gene were significantly associated with susceptibility to BTB in Chinese Holstein cows and that haplotypes TGGACA and CAGACA could be used as genetic markers in marker-assisted breeding programs for breeding cows with high resistance to BTB.

## Introduction

Bovine tuberculosis (BTB) is a chronic disease of cattle caused by *Mycobacterium bovis* [1], a member of the *Mycobacterium tuberculosis* complex [2]. BTB causes direct economic losses in developing countries, because of deficiencies in preventive and control measures [3]. Different occurrence of human TB in different countries and families imply that genetic factors may play a vital role in the TB infection [4]. A previous study reported that among those infected with Mycobacterium tuberculosis population, Only 10% of them will develop to active disease during their life time [5].

Two major candidate genes have been identified to be associated with human TB, the HLA gene family and non-HLA gene. Previous studies have revealed that two isotypes of the HLA gene, HLA-DRB1*04 and HLA-DQB1*0201, are associated with TB in Chinese Kazakh [5], and have identified 17 polymorphic variants of non-HLA gene (NRAMP1, VDR, IL-10, MBP, IFN-γ, TNF-α, MMIF, BTNL2, IL-12, IL-12R, IL-10, RANTES, CCL3, MCP-1,NOS2, CR1,and NOD2) [6–15]. Song [16] et al. found that genotype GG or GA had relative greater risk than those with genotype AA at A1980G site in the TLR6 gene of Holstein cattle. Studies in humans have reported multiple genetic factors and polymorphisms in a number of different genes to be associated with susceptibility to TB [17]. Pinedo et al. studied the potential association of three polymorphisms in the CARD15/NOD2 gene with paratuberculosis infection in cattle and reported that the polymorphic loci SNP2197/C733R was significantly associated with susceptibility to paratuberculosis (PTB) [18]. It has been shown that SNP rs43710290 in the NOD2 gene is associated with PTB, and genotype C/T has a higher distribution in animals infected by *Mycobacterium avium* subsp. *paratuberculosis* (MAP) [19]. In another study, SNP 521 G>A in the NOD2 exon4 showed a significant association with the fecal culture status of MAP [20]. SNP associations to disease susceptibility may indicate that these genetic loci are involved in the immune response to disease through the process of bacterial infection and bacterial replication [21]. Previous studies also revealed the association between CARD15 gene polymorphisms and other human diseases; for instance, a study showed that NOD2 gene variations contribute to Crohn’s disease susceptibility by altering the recognition of microbial pathogens, which changes the activation of NF-kB [22]. Moller et al. showed that R702W, G908R, and 1007fs in the CARD15 gene are not associated with TB in a South African population [23]. Austin et al. identified 11 polymorphic loci in the CARD15 gene, six of which lead to amino acid transitions; pro268ser and Arg702trp are protective to TB, whereas Ala725Gly is associated with TB susceptibility in African Americans [24].

Wang et al. performed a meta-analysis to assess the association between NOD2 polymorphisms and TB risk and revealed that Arg702trp was a protective factor for TB, while Arg587Arg and Gly908Arg polymorphisms were not associated with susceptibility to TB [25]. To the best of our knowledge, there are no previously reported studies on the relationship of genetic variations in the CARD15 gene with susceptibility to BTB. The objective of this study was to identify and evaluate associations of single-nucleotide polymorphisms (SNPs) in the CARD15 gene with susceptibility to BTB in Chinese Holstein cows.

## Materials and Methods

### Ethics

The Animal Care and Use Committee of the Center for Animal Disease Control and Prevention (CADC) of Yunnan province deemed it unnecessary to obtain ethical clearance for this study as the blood samples used only for DNA extraction. All procedures carried out on animals were permitted by the Institutional Animal Care and Use Committee of Yunnan Agricultural University.

### TB test and case-control definition

In total, 201 Chinese Holstein cows were selected from Shiling, Anling, and Niliang,dairy cattle farms in Kumming City; Eryuan, Qiangying, and Dengchan dairy cattle farms in Dali City; and Chenjiang, Nanhua, and Jianchan dairy cattle farms in Yuxi City. All animals were considered to be equally exposed to *M*. *bovis*, because these three cities are not far from each other, and according to local epidemiological data, all 9 farms have similar incidence (5%) of BTB and had been exposed to *M*. *bovis* more than 1 year before testing. All samples were collected in less than 1 month, from the same breeding house, and from animals of the same age. BTB was tested using both the comparative cervical tuberculin (CCT) test and interferon gamma (IFN)-γ assay to avoid any false-positive or false-negative results. A cow was considered to be positive in BTB (case), if it yielded positive results in one or both tests, and showed up symptoms for emaciation, cough, dyspnea, and lymphadenectasis, while it was considered negative only if it yielded negative results in both tests and without clinical symptoms. Out of 201 cows, 103 were positive (cases) and 98 were negative (controls).

### Genomic DNA Extraction

All blood samples were taken as part of routine care and venous blood with anticoagulant was stored at -80˚C until DNA extraction. Genomic DNA was extracted with TaKaRa MiniBEST Whole Blood Genomic DNA Extraction Kit (TaKaRa Bio, Japan) and DNA samples were stored at -20˚C. The CARD15/NOD2 gene (Gene ID: 444867), located on chromosome 18 (AC_000175.1) was used to design 21 primer pairs using Oligo Primer Analysis software v.5 (Molecular Biology Insights, Inc., USA). All primers were synthesized by the Beijing Genomics Institute (BGI), China (S1 Table).

### PCR and RFLP-PCR analysis

PCR reaction was conducted using 0.5 μL TaKaRa Taq DNA polymerase (5 U·μL^-1^), 3 μL 10× PCR buffer, 2.5 μL dNTP mixture, 0.5 μL upstream primer (10 pmol·μL^-1^), 0.5 μL downstream primer (10 pmol·μL^-1^), 2 μL template DNA (10 pmol·μL^-1^), and 21 μL sterile purified water. PCR conditions consisted of an initial denaturation at 95°C for 5 min, followed by 30 cycles of denaturation at 94°C for 45 s, annealing at 51–60°C for 45 s, and extension at 72°C for 1.5 min and a final extension at 72°C for 8 min. Reaction products were separated on 1% agarose gel. Enzyme restriction digestion reaction was conducted using 2 μL 10× buffer, 1 μL *Sty*I restriction enzyme, 13 μL sterile purified water, and 4 μL template DNA (10 pmol·μL^-1^) in a water bath at 37°C for 3 to 4 h. Reaction products were separated on 4% Sepharose gel (Sigma-Aldrich, USA) and compared with a 2000-bp DNA Ladder Marker (TaKaRa, Japan). PCR amplification products were sequenced by BGI and then analyzed using Lasergene software (DNASTAR, USA). DNA sequences were imported into SeqMan program of DNASTAR and the crest of each nucleotide was analyzed. Each nucleotide with two crests was considered to be a polymorphic locus (SNP).

### Statistical analysis

Data were analyzed using SPSS v. 13.0 (IBM, USA). Allele and genotype frequencies were compared between cases and controls using chi-square test or Fisher’s exact test. The odds ratios (ORs) and 95% confidence intervals (CIs) were estimated to quantitatively assess the degree of association between polymorphisms and BTB. Hardy-Weinberg equilibrium (HWE) was tested for each SNP. Linkage disequilibrium analysis was performed using SNPStats software and D’>0.75 means two SNPs are significantly linked [26]. SNP haplotypes were assigned using the online software platform SHEsis (www.analysis2.bio-x.cn/myanalysis.php). Differences were considered statistical significant at p < 0.05.

## Results

**Fig 1:**
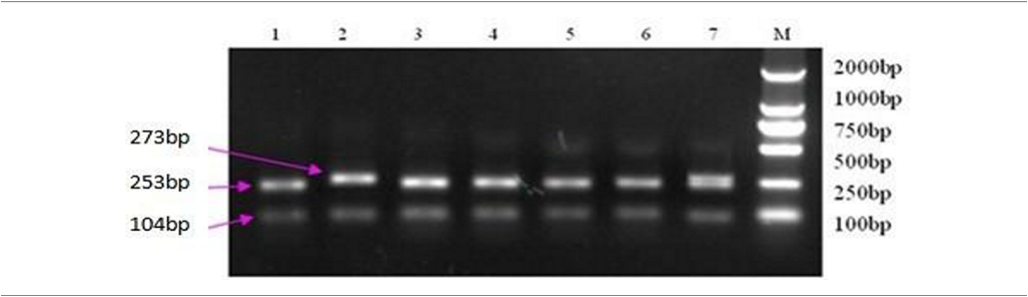
Results of *Sty*I-enzyme digestion at loci E10 (+109). M lane is the DL2000 marker, bands of 273, 253, 104, 20 bp for the A/G genotype in lane 1 and 20bp is invisible in the figure, bands of 253, 104, 20bp for G/G genotype in lanes 2,3, 4, 5, 7, bands of 273, 104 bp for A/A genotype in lane 6.

The CARD15 gene in dairy cows includes 12 exons and 11 introns. It is located on Chromosome 18 and encodes 1013 amino acids. Of the 21 primers’ product that were designed in this study, 10 has polymorphisms and 13 SNPs were detected (Table 1). Three polymorphic loci could lead to an amino acid change; 208 (G/A) could change arginine (R) to histidine (H), 1644 (G/A) could change valine (V) to isoleucine (I), and 1648 (G/A) could change glycine (G) to glutamic acid (E). All other polymorphic loci [E2 (+718)(A/G), E2 (+1016)(A/G), E3 (+1303)(G/T), E4 (-37)(T/C), 1799(C/T), E5 (-68)(T/G), E7 (+349)(C/T), E9 (-257)(A/G), E10 (+107)(A/G), and 1531(A/C)] could not lead to an amino acid change. Of these 13 SNPs, E2 (+718)(A/G), E2 (+1016)(A/G), E3 (+1303)(G/T), E7 (+349)(C/T), and E10 (+107)(A/G) and 1531(A/C) were deposited in Genbank. Five SNPs, E4 (-37), 208, 1644, 1648, and 1799, that had not been studied previously and E10 (+107)(A/G)), which could be recognized by the restriction enzyme *Sty*I, were selected and analyzed. Amplified fragment length of E10 (+107) was 377 bp and StyI enzyme recognized two digestion sites on this fragment (Fig 1). Fragment bands of 20 bp, 253 bp, and 104 bp were corresponded to genotype G/G; fragment bands of 273 bp and 104 bp were corresponded to genotype A/A; and fragment bands of 20 bp, 104 bp, 253 bp, and 273 bp were corresponded to genotype A/G The band of 20bp fragment was not always visible on the gel.

**Table 1:** Single nucleotide polymorphisms (SNP) in the CARD15 gene of Holstein cattle.

Primers	Polymorphic loci	Base mutation	Location	Reference SNP ID number	Changes in amino acids
F4/R4	E2(+718)	A/G	Intron2	133993580	-
F5/R5	E2(+1016)	A/G	Intron2	110406852	-
F8/R8	E3(+1303)	G/T	Intron3	110485603	-
F9/R9	E4(-37)	T/C	Intron3	-	-
	208	G/A	Exon4	-	R-H
F11/R11	1644	G/A	Exon4	-	V-I
	1648	G/A	Exon4	-	G-E
	1799	C/T	Exon4	-	-
F12/R12	E5(-68)	G/T	Intron4	-	-
F13/R13	E7(+349)	C/T	Intron7	110864224	-
F15/R15	E9(-257)	A/G	Intron8	-	-
F16/R16	E10 (+107)	A/G	Intron10	132724082	-
F20/R20	1531	A/C	Exon12	43710289	-

The results of allele and genotype frequency analysis suggested that the frequency of six SNPs was more than 1%, and all p values were higher than 0.05. Therefore, the distribution of genotype frequency followed the Hardy-Weinberg balance rule in cases and controls and was representative for its group (Table 2).

**Table 2:** Genotype and allele distribution of CARD15 gene polymorphisms (SNP) in cases and controls.

	Group	Genotype count (Frequencies)			Allele count (Frequencies)		HWE *(P value)
		CC	TT	CT	T	C	
E4 (-37)	Case	0(0)	87(0.84)	16(0.16)	190(0.92)	16(0.08)	1
	Control	0(0)	97(0.99)	1(0.01)	195(0.99)	1(0.01)	1
		AA	GG	AG	G	A	
208	Case	0(0)	87(0.84)	16(0.16)	190(0.92)	16(0.08)	1
	Control	0(0)	97(0.99)	1(0.01)	195(0.99)	1(0.01)	1
		GG	AA	AG	A	G	
1644	Case	92(0.89)	0(0)	11(0.11)	11(0.05)	195(0.95)	1
	Control	97(0.99)	0(0)	1(0.01)	1(0.01)	195(0.99)	1
		GG	AA	AG	A	G	
1648	Case	0(0)	90(0.87)	13(0.13)	193(0.94)	13(0.06)	1
	Control	0(0)	98(1)	0(0)	196(1)	0(0)	1
		CC	TT	CT	T	C	
1799	Case	93(0.9)	0(0)	10(0.1)	10(0.05)	196(0.95)	1
	Control	98(1)	0(0)	0(0)	0(0)	196(1)	1
		AA	GG	AG	G	A	
E10 (+107)	Case	3(0.03)	57(0.55)	43(0.42)	157(0.76)	49(0.24)	0.18
	Control	1(0.01)	92(0.94)	5(0.05)	189(0.96)	7(0.04)	0.1

* HWE: Hardy-Weinberg equilibrium

Out of six Single nucleotide polymorphisms (SNPs), 1799 had the highest homozygosity level (0.96), while E10 (+107) had the highest effective allele number (1.32). All polymorphism information content (PIC) values were less than 0.25, signifying the low polymorphism of six SNPs (Table 3).

**Table 3:** Homozygosity (Ho), heterozygosity (He), effective allele number (Ne), and polymorphic information content (PIC) of CARD15 gene polymorphisms (SNP).

SNP	Ho	He	Ne	PIC
E4(-37)	0.92	0.08	1.09	0.07*
208	0.92	0.08	1.09	0.07*
1644	0.94	0.06	1.06	0.06*
1648	0.94	0.06	1.06	0.06*
1799	0.96	0.04	1.04	0.04*
E10(+107)	0.76	0.24	1.32	0.21*

PIC>0.5 stands for high polymorphism; 0.25<PIC<0.5 as moderate polymorphism; PIC<0.25 stands for low polymorphism.

* Low polymorphism (PIC<0.25).

Statistical results showed that genotype frequencies of E4 (-37), 208, 1644, 1648, and 1799 were significantly different between cases and controls (P<0.05) (Table 4). Genotype C/T frequency of E4 (-37), genotype A/G frequency of 208, and genotype G/A frequency of 1644 in cases were significantly higher than those in controls (1%) and ORs (95% CI) were lower than 1, suggesting that these genotypes were probably susceptible to BTB. According to minimum Akaike information criterion, genotype A/G and A/A frequencies of E10 (+107) were higher in cases than in controls, and ORs (95% CI) were lower than 1, suggesting that these genotypes were probably susceptible to BTB.

**Table 4:** Associations between E4 (-37), 208, 1644, 1648, 1799, E10 (+107), and bovine tuberculosis in Chinese Holstein cows.

SNP	Model	Genotype	Cases	Controls	OR (95%CI)*	P-value	AIC**	BIC***
E4 (-37)	-	T/T	87(84.5%)	97(99%)	1.00	0.0001	266.1	272.7
		C/T	16(15.5%)	1(1%)	0.06(0.01–0.43)			
208	-	G/G	87(84.5%)	97(99%)	1.00	0.0001	116.1	272.7
		A/G	16(15.5%)	1(1%)	0.06(0.01–0.43)			
1644	-	G/G	92(89.3%)	97(99%)	1.00	0.0018	272.8	279.4
		G/A	11(10.7%)	1(1%)	0.09(0.01–0.68)			
1648	-	A/A	90(87.4%)	98(100%)	1.00	<0.0001	264.3	270.9
		G/A	13(12.6%)	0(0%)	0.00(0.00-NA)			
1799	-	C/C	93(90.3%)	98(100%)	1.00	0.0002	268.7	275.3
		C/T	10(9.7%)	0(0%)	0.00(0.00-NA)			
E10(+107)	Co-dominant	G/G	57(55.3%)	92(93.9%)	1.00	<0.0001	240.8	250.7
		A/G	43(41.8%)	5(5.1%)	0.07(0.03–0.19)			
		A/A	3(2.9%)	1(1%)	0.21(0.02–2.03)			
	Dominant	G/G	57(55.3%)	92(93.9%)	1.00	<0.0001	239.5	246.1
		A/G-A/A	46(44.7%)	6(6.1%)	0.08(0.03–0.20)			
	Recessive	G/G-A/G	100(97.1%)	97(99%)	1.00	0.33	281.6	288.2
		A/A	3(2.9%)	1(1%)	0.34(0.04–3.36)			
	Over-dominant	G/G-A/A	60(58.2%)	93(94.9%)	1.00	<0.0001	241	247.6
		A/G	43(41.8%)	5(5.1%)	0.08(0.03–0.20)			
	Log-additive	-	-	-	0.11(0.05–0.25)	<0.0001	243.2	249.8

* OR stands for odd ratio and CI stands for confidence intervals; OR<1 and P<0.05 mean that all SNP are statistically significant.

** AIC stands for Akaike information criterion.

*** BIC stands for Bayesian information criterion.

Linkage Disequilibrium Analysis was performed for above 6 SNPs and showed that E4(-37)and 208 (D’ = 0.9980),E4(-37) and 1644 (D’ = 0.9588),208 and 1644(D’ = 0.9588),1644 and 1799 (D’ = 0.8960),1648 and 1799(D’ = 0.8961) are tightly linked. SNP E10(+107) and E4(-37),E10(+107) and 208 (D’ = 0.5225) are common linked (S1 Fig). We identified and analyzed haplotypes based on six polymorphic SNPs and discarded those with frequency of occurrence less than 0.01. The distribution of two haplotypes, TGGACA (OR = 1.00) and CAGACA (OR < 1), was significantly different between cases and controls (Table 5), and both were significantly associated with BTB, suggesting that these haplotypes were probably susceptible to BTB.

**Table 5:** Haplotype analysis of E4 (-37), 208, 1644, 1648, 1799 and 10(+107) in the CARD15 gene.

Haplotype	Frequency	Cases	Controls	P-value	OR(95%CI
TGGACG	0.8163	0.6673	0.9592	-	1.00
TGGACA	0.0839	0.1463	0.0306	0.0001*	0.15 (0.06–0.37)
CAGACA	0.0259	0.0413	0.0051	0.0058*	0.05 (0.01–0.41)
TGGGTG	0.0141	0.0332	0	-	-
CAGACG	0.0141	0.0332	0	-	-
TGAACA	0.0138	0.025	0	-	-
TGAACG	0.0112	0.019	0.0051	-	-

Asterisk (*) denotes statistical significance, P<0.05.

* OR stands for odd ratio and CI stands for confidence intervals.

## Discussion

The occurrence and development of TB are affected by many factors, including pathogen, host immune level, and environment. Among these factors, susceptible genes play an important role [27–28]. The CARD15 gene is a pattern recognition receptor (PRR) for bacterial lipopolysaccharides (LPS). LPS release inflammatory mediators that recognize the muramyl dipeptide and activate NF-kb that controls transcription of tumor necrosis factor-α, interferon-γ, interleukin-1β, and interleukin-12 [4]. The CARD15 protein participates in immune response of cells and plays an important role in antibodies production. A CARD15 gene mutation that leads to changes in protein structure could be a risk factor of TB [4]. The NRAMP1, VDR, MBP, TLR, and NRAMP1 genes are able to regulate intracellular pathogen proliferation by altering the internal environment of the phagolysosome [5]. The vitamin D nuclear receptor assists macrophages to restrain the development of TB in cells [4]. Toll-like Receptors and nucleotide-binding oligomerization domain-like receptors play a protective role in TB [4].

CARD15 gene variations are associated with many diseases such as TB, Crohn’s disease, and paratuberculosis. A previous study showed that the pro268ser, Arg702Trp SNPs of the CARD15 gene are associated with human TB [23]. Additionally, the reported results on TB candidate genes have been inconsistent, due to the different genetic and environmental conditions in the studies or interactions between haploid variations and genetic mechanisms, and R702W and 1007fs polymorphisms in the CARD15 gene have been reported as significantly associated with Crohn’s disease [29–33].

The CARD15/NOD2 gene is our first step in exploring the influence of genetic factors on BTB. We identified 13 SNPs in the NOD2 gene using 21 primers and selected 6 SNPs for further analysis. In our study, E4 (-37), 208, 1644, 1648, and 1799 had two genotypes, and the genotype frequency of E4 (-37) (C/T), 208 (A/G), 1644 (G/A), 1648 (A/G), and 1799 (A/G) was significantly different between cases and controls. The CARD15 protein, which is used as a pattern recognition receptor (PRR) and consists of leucine-rich repeats (LRR), a NACHT domain, and two caspase recruitment domains (CARD), plays a vital role in innate and acquired immunity. SNP 208, 1644, and 1648 are all located in exon 4 of the CARD15 gene and linked to three missense mutations in the LRR domain. E10(+107) was the only SNP that had three genotypes, A/A, G/G, and A/G; therefore, we analyzed E10(+107) using different models. In the co-dominant model, A/A, G/G, and A/G were all dominant, and the frequency of genotypes was significantly different between cases and controls (P < 0.0001). G/G had a higher distribution in controls (93.9%) than in cases (55.3%), whereas A/G and A/A had a higher distribution in cases (41.8% and 2.9%, respectively) than in controls (5.1% and 1%, respectively). In the dominant model, G/G and A/G-A/A were dominant and had significant differences between cases and controls (P < 0.0001), while in over-dominant model, G/G-A/A and AG were dominant and also had significant differences between cases and controls (P < 0.0001). In the recessive model, no differences (P = 0.33) were observed for G/G-A/G and A/A between cases and controls, while log-additive analysis was not suitable for this study. The A/G or A/A genotypes of SNP E10(+107) had greater relative risk than G/G. However, before these results are used in marker-assisted selection for BTB resistance in dairy cattle, the third genotype of SNP E4 (-37), 208, 1644, 1648, and 1799 needs to be detected using larger sample populations. Haplotype analysis suggested that the frequency of TGGACA and CAGACA was significantly different between cases and controls (P < 0.05). Cattle with these two haplotypes have greater possibility to be infected and attacked by *M*. *bovis*; however, further study is needed to elucidate the role of TGGACA and CAGACA in the process of infection.

## Supporting Information

S1 Fig Linkage disequilibrium analysis of SNPs in the CARD15 gene. (DOCX)

S1 Table Primers of the CARD15 gene amplification. (DOCX)
