# Supplementary material for: CARD15 Gene Polymorphisms Are Associated with Tuberculosis Susceptibility in Chinese Holstein Cows
Source: PLoS One. 2015 Aug 5;10(8):e0135085. doi: 10.1371/journal.pone.0135085 (PMC4526225; doi:10.1371/journal.pone.0135085)
Supplement: S1 Fig — (DOCX) [file pone.0135085.s001.docx]

**Supporting Information**

**S1 Figure:** Linkage disequilibrium analysis of SNPs in the CARD15 gene


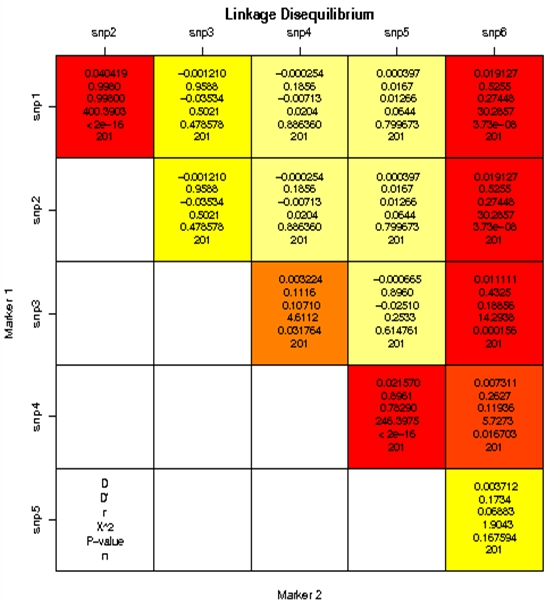


There is significant correlation between two of these six SNPs.
