# Supplementary material for: CARD15 Gene Polymorphisms Are Associated with Tuberculosis Susceptibility in Chinese Holstein Cows
Source: PLoS One. 2015 Aug 5;10(8):e0135085. doi: 10.1371/journal.pone.0135085 (PMC4526225; doi:10.1371/journal.pone.0135085)
Supplement: S1 Table — (DOCX) [file pone.0135085.s002.docx]

**Supporting Information**

S1 Table: Primers of CARD15 amplification.

| Primers | Sequences（5’-3’） | length | location | Size | annealing temperature(℃) |
| --- | --- | --- | --- | --- | --- |
| CARD15-F1 | GGT GGG GTC TCT CTG TGC TAG TC | 23 | 5’ region | 802 | 55 |
| CARD15-R1 | AGG AAA CTG AGG CAG CCA GGT A | 22 | 5’ region |  |  |
| CARD15-F2 | CTG TGT TCC TTT CTG GTT TAC AGTG | 25 | Exon1 | 794 | 53 |
| CARD15-R2 | AAC CCA GGT GAA GTG AAA CTC GT | 23 | Intron1 |  |  |
| CARD15-F3 | TTA AAC CAG GCA TGA CGC ATA G | 22 | Intron1 | 830 | 51 |
| CARD15-R3 | GAG GAG GAC TAT GAC CCA CAT CT | 23 | Intron2 |  |  |
| CARD15-F4 | GGT AAG CAC TTC CCT CTA AGC AC | 23 | Intron2 | 757 | 55 |
| CARD15-R4 | TGC TTA CTG TGT GCT GGG TAC TG | 23 | Intron2 |  |  |
| CARD15-F5 | TTC TCT GTG GAA GCT CTC ATT AC | 23 | Intron2 | 849 | 51 |
| CARD15-R5 | GTA CAA GGT TCT CAG CAC AAT AAG | 24 | Intron2 |  |  |
| CARD15-F6 | TAA ACC CAG GGC TTT GAC TAA T | 22 | Intron2 | 785 | 51 |
| CARD15-R6 | CTA TAA ACC TGC AAG AAT GGG AA | 23 | Intron2 |  |  |
| CARD15-F7 | TGG GGA TTT CAG AGA CTC GAA G | 22 | Intron2 | 798 | 53 |
| CARD15-R7 | ACC CCA CAG ACT GTA GCC TAC C | 22 | Intron3 |  |  |
| CARD15-F8 | GTA GGG GAG GAT GGA TCT GGT T | 22 | Intron3 | 762 | 51 |
| CARD15-R8 | CAA CCA GGT ACT GAC CCA TTT GT | 23 | Intron3 |  |  |
| CARD15-F9 | ACC ACC ACT TCT CTG TCC CAT AG | 23 | Intron3 | 809 | 55 |
| CARD15-R9 | GAA GGT TGA AGA GCA GAC TCT GG | 23 | Exon4 |  |  |
| CARD15-F10 | CCT CTT AAC CTT TGA TGG CTT TG | 23 | Exon4 | 782 | 54 |
| CARD15-R10 | GCC TGT GAT CTT GGA AGA GAT GT | 23 | Exon4 |  |  |
| CARD15-F11 | TGC TTC TTT GCT GCA TTC TAC CTC | 24 | Exon4 | 796 | 55 |
| CARD15-R11 | CTA AGG TGC AGA CTG ATG AGG GAT | 24 | Intron4 |  |  |
| CARD15-F12 | CCC ACC GAG CGT ATG ACA TTA GTA | 24 | Intron4 | 806 | 55 |
| CARD15-R12 | TCT TCA GTC CTG GGA GCT AAA GGT | 24 | Intron6 |  |  |
| CARD15-F13 | GAC TGT TGT GGT TCC TGC TCC TAC | 24 | Intron6 | 816 | 53 |
| CARD15-R13 | TCT CAC CCC AAG CTA CAC ATC ATC | 24 | Intron7 |  |  |
| CARD15-F14 | GCA CTG GGT GGA CCT CTT AAC T | 22 | Intron7 | 788 | 51 |
| CARD15-R14 | CAA TGG CTC CGT GGA TAA AGA A | 22 | Intron7 |  |  |
| CARD15-F15 | ATT TCC TCC TCC AGG GGA TCT T | 22 | Intron7 | 854 | 53 |
| CARD15-R15 | ACA CAC ACA TCA GCT TCC ACA GA | 23 | Intron9 |  |  |
| CARD15-F16 | CAC ATG GGT TCA TCT TTA CTG G | 22 | Intron9 | 890 | 53 |
| CARD15-R16 | GCC TTT TTA TCC CTC TAT CCT CA | 23 | Intron10 |  |  |
| CARD15-F17 | TCA TCA CAG AGC CCT GAG CTA A | 22 | Intron10 | 796 | 52 |
| CARD15-R17 | TTC CCT TTT TAA CCA CAC TGC C | 22 | Intron11 |  |  |
| CARD15-F18 | TGC ACA ATT CTG AGA AAT GTC AC | 23 | Intron11 | 743 | 53 |
| CARD15-R18 | AGG ATT GAA TCT TGG CAA GAA GT | 23 | Exon12 |  |  |
| CARD15-F19 | CAA CCC AAT AGG TCA CCT TTG TTC | 24 | Exon12 | 716 | 51 |
| CARD15-R19 | CAG AGT TCA GTT CAC AGG CTT CA | 23 | Exon12 |  |  |
| CARD15-F20 | CCA GAA CTA TCT TCA TTG TGA CC | 23 | Exon12 | 784 | 52 |
| CARD15-R20 | CAG GGA TGG AGT AAG AGA TTG AG | 23 | 3-UTR |  |  |
| CARD15-F21 | GTT AAA CCT TTG GGA ACA ACT CT | 23 | Exon12 | 532 | 51 |
| CARD15-R21 | CTC TAA ACT GAA GCC AAA TGT GT | 23 | Exon12 |  |  |

Selection information of case and control

In our study, 201 Chinese Holstein cows were selected from Shiling (case 12,control 11), Anling Xingdian (case 12,control 11), and Niliang (case 11, control 10),dairy cattle farms in Kumming City; Eryuan (case 12,control 11), Qiangying (case 12,control 11), and Dengchan (case 10, control 11) dairy cattle farms in Dali City; and Chenjiang (case 12,control 11), Nanhua (case 12,control 11), and Jianchan (case 10, control 11) dairy cattle farms in Yuxi City.All samples were from same breeding house and from animals of the same age for 8.
